# Dual-Action Tocilizumab-Conjugated Cisplatin Nanoparticles Overcome Chemoresistance and Metastasis in Non-Small-Cell Lung Cancer

**DOI:** 10.3390/pharmaceutics17070945

**Published:** 2025-07-21

**Authors:** Yin Wang, Fanyu Wu, Tan Yang, Bin Li, Han Wang, Peng Ye, Weijie Li

**Affiliations:** 1 Department of Pharmacy, Tongji Hospital, Tongji Medical College, Huazhong University of Science and Technology, Wuhan 430030, China; 2 Department of Laboratory Medicine, Wuhan Hospital of Traditional Chinese and Western Medicine, Tongji Medical College, Huazhong University of Science and Technology, Wuhan 430030, China; wangyin820714@126.com; 3 Department of Geriatrics, The Second Affiliated Hospital of Chongqing Medical University, Chongqing 400010, China; wufanyu92@163.com; 4 School of Pharmacy, Tongji Medical College, Huazhong University of Science and Technology, Wuhan 430030, China; yangtan0120@hust.edu.cn (T.Y.); libin@youdubio.com (B.L.); 15971506312@163.com (H.W.); 5 Department of Pharmacy, Renmin Hospital of Wuhan University, Wuhan 430030, China

**Keywords:** cisplatin, interleukin 6, tocilizumab, epithelial–mesenchymal transition, cancer stem cells

## Abstract

**Background/Objectives:** Cisplatin remains a cornerstone chemotherapeutic agent for non-small-cell lung cancer (NSCLC) treatment, yet its clinical utility is substantially limited by acquired resistance and the inadequate suppression of tumor metastasis. Emerging evidence implicates interleukin 6 (IL-6) as a critical mediator of chemoresistance through cancer stem cell (CSC) enrichment and metastasis promotion via epithelial–mesenchymal transition (EMT) induction, ultimately contributing to cisplatin therapy failure. This study sought to address these challenges by designing a nanoplatform with two innovative aims: (1) to achieve active tumor targeting through binding to the IL-6 receptor (IL-6R), and (2) to concurrently inhibit IL-6-mediated chemoresistance signaling pathways. **Methods:** A lipid–polymer hybrid nanoparticle (LPC) encapsulating cisplatin was synthesized and subsequently surface-functionalized with tocilizumab (TCZ), a monoclonal antibody that targets IL-6R. The therapeutic efficacy of this TCZ-modified nanoparticle (LPC-TCZ) was assessed through a series of in vitro and in vivo experiments, focusing on the inhibition of EMT, expression of CSC markers, tumor growth, and metastasis. **Results:** Systematic in vitro and in vivo evaluations revealed that LPC-TCZ synergistically attenuated both EMT progression and CSC marker expression through the targeted blockade of IL-6/STAT3 signaling. This multimodal therapeutic strategy demonstrated superior tumor growth inhibition and metastatic suppression compared to conventional cisplatin monotherapy. **Conclusions:** Our findings establish a nanotechnology-enabled approach to potentiate cisplatin efficacy by simultaneously countering chemoresistance mechanisms and metastatic pathways in NSCLC management.

## 1. Introduction

Lung cancer persists as a leading global health crisis, ranking second in cancer-related mortality worldwide with an estimated 2.2 million new cases annually [1]. This malignancy’s pathogenesis is inextricably linked to tobacco exposure (accounting for 85% of cases), environmental carcinogens, and occupational hazards [2]. Non-small-cell lung cancer (NSCLC), constituting 85% of pulmonary malignancies [3], manifests as histologically distinct subtypes, including adenocarcinoma, squamous cell carcinoma, and large-cell carcinoma [4]. Despite therapeutic advances, over 70% of NSCLC patients present with locally advanced or metastatic disease at diagnosis, resulting in 5-year survival rates below 15% [5,6]. Platinum-based chemotherapy regimens combining cisplatin (cis-diamminedichloroplatinum II—CDDP) with taxanes or vinca alkaloids remain the first-line systemic therapy, though their clinical utility is severely constrained by two fundamental limitations [7,8]. CDDP exerts cytotoxic effects through DNA cross-linking, disrupting replication and transcription mechanisms; however, its non-selective biodistribution causes dose-limiting nephrotoxicity, neurotoxicity, and myelosuppression [9,10,11]. While nanoparticle-mediated delivery systems have emerged to enhance tumor accumulation and reduce off-target toxicity, a more insidious challenge persists—acquired chemoresistance [12,13]. Chronic CDDP exposure induces epithelial–mesenchymal transition (EMT) and enriches cancer stem cell (CSC) populations via STAT3-mediated pathways, creating treatment-refractory tumors with enhanced metastatic potential [14,15,16,17]. This biological transformation establishes a self-reinforcing cycle of therapeutic failure, demanding innovative strategies to simultaneously overcome chemoresistance and metastasis.

The interleukin 6 (IL-6) signaling axis has recently been implicated as a master regulator of this malignant progression. Within the tumor microenvironment (TME), stromal cells and malignant clones secrete IL-6, which activates gp130/JAK/STAT3 cascades through IL-6 receptor (IL-6R) binding [18,19]. This pathway orchestrates chemoresistance by upregulating EMT markers (N-cadherin, Twist, and Snail) while suppressing E-cadherin and concurrently enhancing CSC plasticity via Oct-4/Sox-2/Nanog overexpression [20,21,22]. Preclinical evidence confirms that IL-6 pathway inhibition resensitizes tumors to CDDP, yet clinical translation requires precise spatial control of the IL-6R blockade within the TME [23,24,25]. Tocilizumab (TCZ), a humanized anti-IL-6R monoclonal antibody that has been clinically approved for autoimmune disorders, exhibits emerging anti-tumor activity across multiple malignancies [26,27]. While previous nanoscale TCZ formulations focused on rheumatoid arthritis treatment through passive adsorption [28], no studies have exploited TCZ’s tumor-targeting potential via covalent conjugation or investigated its synergy with chemotherapeutics. We hypothesized that engineering TCZ-conjugated nanoparticles could achieve dual therapeutic objectives: (1) active tumor targeting through IL-6R binding, and (2) the simultaneous blockade of IL-6-driven chemoresistance pathways.

Herein, we present a rationally designed lipid-coated cisplatin nanovesicle (LPC) functionalized with site-specific TCZ conjugation (LPC-TCZ) using EDC/NHS chemistry. This novel nanoarchitecture provides four critical advances. First, the lipid bilayer enhances CDDP solubility while enabling TCZ orientation control for optimal receptor engagement. Second, TCZ-mediated active targeting synergizes with enhanced permeability and retention (EPR) effects for tumor-selective accumulation. Third, the localized IL-6R blockade disrupts STAT3 phosphorylation, reversing EMT and CSC phenotypes to resensitize tumors. Fourth, the spatiotemporal coordination of CDDP release and pathway inhibition creates a self-amplifying therapeutic cycle. Through comprehensive in vitro and in vivo validation, we demonstrate that LPC-TCZ not only overcomes CDDP resistance mechanisms but also suppresses metastatic progression. This is a dual functionality that has never before been achieved in NSCLC nanotherapeutics.

## 2. Materials and Methods

### 2.1. Materials

CDDP was obtained from Macklin (Shanghai, China). TCZ was purchased from MedChemExpress (Shanghai, China). Silver nitrate (AgNO_3_), potassium chloride (KCl), nitric acid (HNO_3_), cyclohexane, and n-hexanol were received from Sinopharm Chemical Reagent Co. (Shanghai, China). EDC and NHS were produced by Aladdin Reagent Co. (Shanghai, China). Igepal CO-520 was supplied by Bidepharmatech (Shanghai, China). Coumarin-6,3-(4,5-dimethyl-thiazol-2-yl)-2,5-diphenyl tetrazolium bromide (MTT), Triton-X 100, and 4,6-diamidino-2-phenylindole (DAPI) were acquired from Sigma Aldrich Chemical Co. (St. Louis, MO, USA). 1,2-dioleoyl-sn-glycerol-3-phosphate (DOPA) was obtained from Avanti Polar Lipids Inc., based in Alabaster, AL, USA. Hydrogenated soy phosphatidylcholine (HSPC) was produced by AVT Pharmaceutical Tech Co. Ltd. (Shanghai, China). Cholesterol (CHOL) was obtained from Shanghai Advanced Vehicle Technology Co., Ltd. (Shanghai, China). Distearoyl phosphatidylethanolamine-monomethoxy polyethylene glycol 2000 with a carboxyl (DSPE-mPEG2000-COOH) was purchased from Ponsure (Shanghai, China). All chemicals were used as received without further purification.

### 2.2. Cell Culture and Animals

Procell Life Science and Technology Co., Ltd. (Wuhan, China) supplied the A549/CDDP cells and Ham’s F-12K. Fetal bovine serum (FBS) was obtained from Thermo Fisher Scientific (Chicago, IL, USA). A549/CDDP cells were cultured in Ham’s F-12K supplemented with 10% FBS and 1% penicillin–streptomycin. A549/CDDP cells were cultured in 5% CO_2_ incubators at 37 °C. Female BALB/c nude mice aged 4 to 6 weeks were sourced from Huafukang Bioscience Technology Co. in Beijing, China. The Committee on Ethical Animal Experiments at Huazhong University of Science and Technology approved the animal experiments.

### 2.3. Synthesis of Cis-[Pt(NH_3_)_2_(H_2_O)_2_] (NO_3_)_2_

The synthesis of the cisplatin prodrug, cis-[Pt(NH_3_)_2_(H_2_O)_2_](NO_3_)_2_, was conducted following the methodology outlined in the existing literature [29]. AgNO_3_ (132.4 mg, 0.78 mmol) and CDDP (120 mg, 0.40 mmol) were dissolved in 2.0 mL of deionized water. The resulting mixture was heated and stirred at 60 °C for 3 h, followed by overnight stirring in dark conditions. Subsequently, the AgCl precipitate was separated via centrifugation at 8000 rpm for 15 min. The concentration of the cisplatin prodrug was quantified using inductively coupled plasma mass spectrometry (ICP-MS, Agilent 7500, Santa Clara, CA, USA).

### 2.4. Synthesis of the LPC Core

The synthesis route of LPCs is illustrated in Figure 2A. First, cyclohexane/Igepal CO-520 (71:29, V:V) and cyclohexane/Triton-X 100/hexanol (75:15:10, V:V:V) were mixed in a 3:1 ratio. Then, 200 μL of Cis-[Pt (NH_3_)_2_(H_2_O)_2_] (NO_3_)_2_ and DOPA (20 mg/mL) were dispersed in 16.0 mL of the mixed oil phase. Another emulsion was prepared by adding 200 μL of KCl (800 mM) in water into 16.0 mL of the mixed oil phase. The two emulsions were combined after stirring for 20 min, and the reaction continued for an additional 30 min. After the reaction, 32.0 mL of ethanol was added and the mixture was stirred for 5 min. To eliminate the cyclohexane and surfactants, the mixture was spun at 13,000 rpm for 15 min. The pellets were thoroughly washed with ethanol three times, dried, and then re-dispersed in 6.0 mL of chloroform before being stored in a glass vial for further modification.

### 2.5. Synthesis of LPCs

To prepare LPCs, 6.0 mL of LPC core and 20 mg HSPC–cholesterol–DSPE–PEG2000-COOH (molar ratio 55:40:5) were combined. After evaporating the chloroform, the residual lipids were dispersed in 2.0 mL of PBS and rehydrated at 60 °C for at least 30 min. Then, the nanoparticles were spun at 12,000 rpm for 15 min before being washed with PBS three times.

### 2.6. Synthesis Characterization of LPC-TCZ NPs

To prepare LPC-TCZ NPs, 6 mg EDC, 6 mg NHS, and 100 μL TCZ solution (5 mg/mL) were added to the appropriate amount of LPC solution. After stirring for 6 h, the nanoparticles were centrifuged at 12,000 rpm for 15 min and washed with PBS three times.

A Zeta PALS zeta potential analyzer from Brookhaven Instruments Corporation (Austin, TX, USA) was used to measure the average particle size and zeta potential of LPCs and LPC-TCZ NPs. A Tecnai G2 20 transmission electron microscope (Tokyo, Japan) and a Talos F200X field emission transmission electron microscope (Thermo Fisher Scientific, Waltham, MA, USA) were used to observe and photograph the morphology.

A SpectrAA-24OFS atomic absorption spectrometer (Varian, Palo Alto, CA, USA) was used to measure the encapsulation efficiency of CDDP. The coupling rate of TCZ was measured using the BCA reagent method with a Synergy HT microplate reader (Biotek, Winooski, VT, USA).

The presence of TCZ on the nanoparticles was verified using Coomassie blue staining and Western blotting. Firstly, the nanoparticles were ultrasonically broken down with Triton-X 100, and then the lipids and proteins of different molecular weights were separated using SDS-PAGE (Bio-Rad Laboratories, Hercules, CA, USA). Finally, the proteins were stained with Coomassie blue.

### 2.7. In Vitro Drug Release

The release profile of CDDP in vitro was studied using the dynamic dialysis method. A dialysis bag containing 20 mg of LPC-TCZ NPs (MWCO 14 kDa) was immersed in 500 mL of PBS solution at 37 °C. The releasing medium was taken out at a given time to undergo ICP-AAS in order to measure cisplatin-releasing content.

### 2.8. Intracellular Localization of LPC-TCZ NPs

To assess cellular internalization of LPC-TCZ, A549 and A549/CDDP cells were incubated with cisplatin LPC or LPC-TCZ (2.5 μg/mL cisplatin-equivalent dose) for 48 h in 24-well plates. Following nitric acid digestion (70% HNO_3_, diluted to 2% final concentration), intracellular cisplatin levels were quantified by ICP-MS (Agilent 7500, Santa Clara, CA, USA).

DAPI staining was used to track the intracellular localization of LPC-TCZ NPs in A549/CDDP cells. The cells (5 × 10^4^ per well) were seeded in 12-well plates. After overnight incubation, the cells were treated with coumarin 6-labeled LPC-TCZ NPs, followed by incubation at 37 °C for 0.5, 1, 2, 4, 8, and 12 h. To observe nanoparticle uptake, the cells underwent three washes with PBS and the cell nuclei stained with 4′,6-diamidino-2-phenylindole (DAPI) after paraformaldehyde fixation. Then, the stained cells were washed with PBS thrice and monitored under an Olympus SZX12 fluorescence microscope (Tokyo, Japan). For flow cytometry analysis, the cells were washed thrice with PBS and then trypsinized and redispersed. Cell suspension was detected using a Becton Dickinson LSRIIflow cytometer (San Jose, CA, USA).

### 2.9. Cytotoxicity Assay

The cytotoxic effects of TCZ, CDDP, LPC NPs, and LPC-TCZ NPs were measured using an MTT assay. A549/CDDP cells were placed in 96-well plates at a density of 1 × 10^4^ cells per well. Following overnight incubation, the cells were treated with 100 μL of culture media containing different formulations. The cells were subsequently incubated for 24 h. Each well received 20 μL of MTT solution (5 mg/mL in PBS) and was incubated for another 4 h. The fluid in the well was sucked out carefully and was followed by the addition of DMSO. The absorbance was recorded with the Synergy HT microplate reader (Biotek, Winooski, VT, USA).

### 2.10. Wound Healing Assay

The migratory ability of cells was measured using a wound healing assay. A549/CDDP cells were seeded in 12-well plates at 1 × 10^5^ cells per well. Once the cell monolayers reached full confluence, they were scratched with a 200 μL pipette tip and rinsed twice with PBS. Subsequently, the cells were incubated with 1 mL serum-free medium containing different formulations (100 μM CDDP). Photographs were taken using an Olympus SZX12 fluorescence microscope (Tokyo, Japan) at 0 h and 24 h. Then, migration distance was calculated.

### 2.11. Inhibition of Multicellular Tumor Spheroids (MCTSs)

A549/CDDP cells were seeded in 96-well ultralow-adsorption plates at a concentration of 500 cells per well. When the diameter of the MCTSs had grown to 400 μm, the tumor spheroids were incubated with different formulations at a CDDP concentration of 100 μM for 3 d. Photographs were taken using the Olympus SZX12 fluorescence microscope (Tokyo, Japan) at 0 d, 1 d, and 3 d, respectively, and the diameter of the cell spheroids was measured.

### 2.12. Western Blot

A549/CDDP cells were placed in 6-well plates at a density of 1 × 10^5^ cells per well. Following overnight incubation, the cells were treated with 2 mL of culture medium containing different formulations at a CDDP concentration of 100 μM for 24 h. Proteins were extracted using RIPA lysis buffer containing 1% protease inhibitor, 1% PMSF, and 2% cocktail. Then, proteins were separated on 12% SDS-PAGE gel and transferred onto PVDF membranes. The PVDF membranes were treated with 5% skim milk in TBS–0.1% Tween 20 (TBST) for an hour and exposed to monoclonal antibodies against JAK1 (1:1000, Proteintech, USA), p-JAK1 (1:1000, Multi Sciences, China), STAT3 (1:3000, Proteintech), p-STAT3 (1:1000, Multi Sciences), E-cadherin (1:2000, Proteintech), N-cadherin (1:2000, Proteintech), vimentin (1:5000, Proteintech), Snail (1:2000, Bioss Antibodies, China), Oct-4 (1:5000, Proteintech), Nanog (1:5000, Proteintech), Sox-2 (1:5000, Proteintech), and β-tubulin (1:3000, Proteintech) overnight at 4 °C. After being washed three times with TBST, the PVDF membranes were transferred to a secondary antibody diluent (1:2000, Proteintech) and incubated for 1 h. The PVDF membranes were washed three times with TBST. Then, the target proteins were linked with an ECL kit and photographed using a GeneGenome5 chemiluminescence system (Syngene, Cambridge, UK).

### 2.13. Anti-Tumor Efficacy Study In Vivo

To evaluate the in vivo tumor therapeutic effect of NPs, an A549/CDDP xenograft model was established. When the tumor volume reached around 200 mm^3^, A549/CDDP-bearing mice were intravenously injected with PBS, CDDP, TCZ, LPC NPs, and/or LPC-TCZ NPs at 2 mg/kg of CDDP or 5 mg/kg TCZ on days 12, 15, 19, and 22 (*n* = 5). Tumor volume and body weight were measured every 2 days following treatment. On day 30, blood was drawn from the eye socket, and the serum was separated to measure kidney or liver biochemical markers such as BUN, CRE, AST, and ALT. Tumors were then collected, weighed, and photographed. Major organs were collected and sectioned into slices for H&E.

### 2.14. Statistical Analysis

The data were collected from three separate experiments and are presented as means ± standard deviation (SD). Statistical significance was assessed using Student’s *t*-test or one-way analysis of variance (ANOVA) using GraphPad Prism version 8.0 (San Diego, CA, USA).

## 3. Results and Discussion

### 3.1. IL-6 Expression Correlates with Lung Cancer Prognosis

Increased IL-6 expression in lung cancer triggers persistent activation of the STAT3 signaling pathway. IL-6 binds to its receptor, inducing phosphorylation and nuclear translocation of STAT3, which promotes the transcription of oncogenic targets (e.g., cyclin D1 and VEGF). This cascade enhances tumor cell proliferation, survival, and metastasis via EMT [30]. Additionally, STAT3 activation fosters an immunosuppressive tumor microenvironment by upregulating PD-L1 and recruiting immunosuppressive cells [31,32]. Clinical evidence links IL-6/STAT3 hyperactivity to advanced stages and poor prognosis. Targeting this axis shows therapeutic potential, but faces challenges due to pathway redundancy and resistance mechanisms [33].

**Figure 1:**
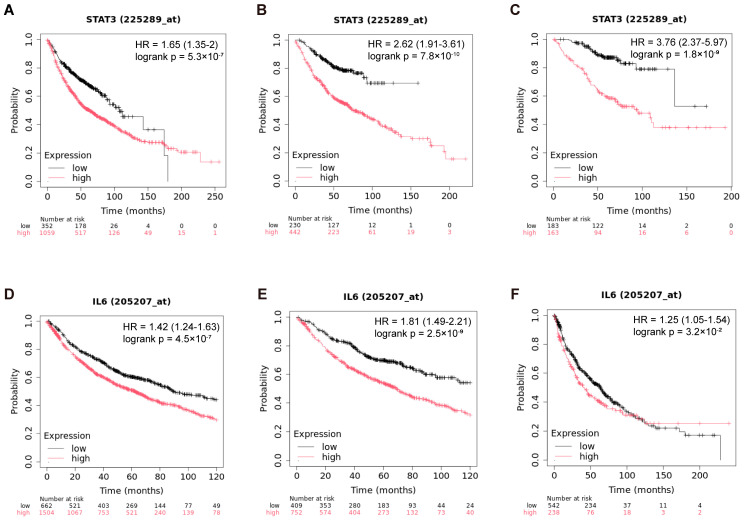
Association of IL-6/STAT3 with survival in advanced lung cancer patients. (**A**) Prognostic correlation of STAT3 protein expression in unclassified lung cancer; (**B**) STAT3 protein expression in the adenocarcinoma cohort; (**C**) STAT3 protein expression in the advanced-stage adenocarcinoma cohort; (**D**) prognostic correlation of IL-6 protein expression in unclassified lung cancer; (**E**) IL-6 protein expression in the adenocarcinoma cohort; (**F**) IL-6 protein expression in the squamous cell carcinoma cohort. Data source: Kaplan–Meier Plotter (https://www.kmplot.com/analysis/index.php?p=home, accessed on 15 December 2024). Hazard ratios (HRs), 95% confidence intervals (CIs), and logrank *p*-values are indicated on the plots.

Using the Kaplan–Meier Plotter database (https://www.kmplot.com/analysis/index.php?p=home, accessed on 15 December 2024), we analyzed the association between IL-6/STAT3 expression and survival in lung cancer patients. Kaplan–Meier analysis revealed that high STAT3 protein expression significantly predicted shorter overall survival (OS) in unclassified lung cancer (Figure 1A), with consistent trends being observed in adenocarcinoma (Figure 1B) and advanced-stage adenocarcinoma (Figure 1C). Similarly, elevated IL-6 expression correlated with reduced OS in both unclassified (Figure 1D–F) and histology-specific subgroups. These findings indicate that IL-6/STAT3 pathway hyperactivation accelerates disease progression and worsens prognosis in advanced lung cancer, highlighting its clinical relevance as a potential therapeutic target.

### 3.2. Characterization of LPC-TCZ NPs

Tumor cells, tumor mesenchymal stem cells, and tumor-related fibroblasts secrete IL-6, leading to manifestation of the EMT phenotype and stem cell characteristics in tumor cells, which results in decreased efficacy of CDDP [34]. Therefore, in this study, the IL-6R neutralizing antibody TCZ was combined with CDDP to reverse the drug resistance of cisplatin in the treatment of non-small-cell lung cancer. However, CDDP exhibits poor lipid solubility and water solubility and has strong systemic toxicity and side effects [29]. The development of a nanoscale drug delivery system not only enhances the water solubility of CDDP but also increases its accumulation at the tumor site through the EPR effect, thereby reducing the toxic side effects of CDDP on normal tissues. A nanosized DOPA package was prepared using the reverse-phase microemulsion method and loaded with cisplatin. Lipid-coated cisplatin nanoparticles were then prepared using the thin-film hydration method, with the introduction of DSPE-mPEG2000-COOH into the phospholipid bilayer of the nanoparticles. PEG was used to increase the long cycle stability of the nanoparticles. Finally, TCZ was conjugated with the nanoparticle surface via EDC/NHS.

**Figure 2:**
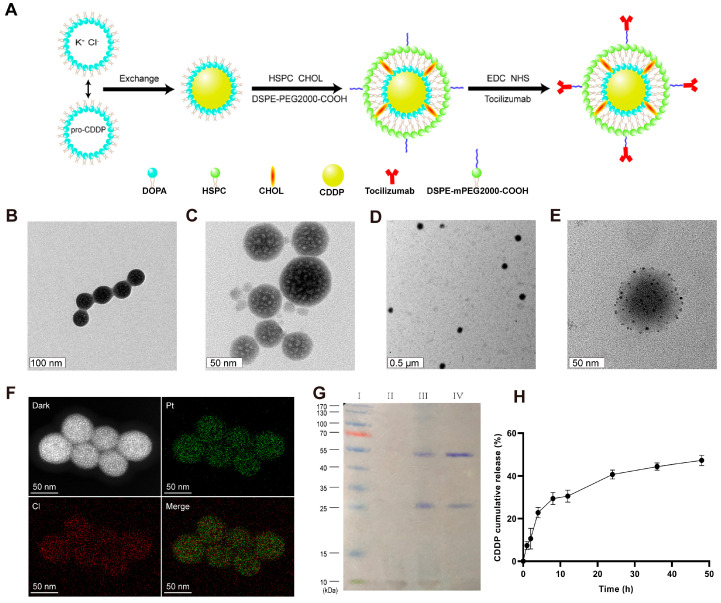
Characterization of LPC-TCZ nanoparticles. (**A**) Schematic representation of the synthesis route for LPC-TCZ nanoparticles. (**B**) TEM images of the LPC core. (**C**–**E**) TEM images of LPC-TCZ nanoparticles. (**F**) Elemental mapping of the LPC core, showing the uniform distribution of platinum and chlorine elements. (**G**) SDS-PAGE image of marker (lane I), LPCs (lane II), LPC-TCZ NPs (lane III), and free TCZ (lane IV). (**H**) In vitro cumulative release curve of cisplatin from LPC-TCZ nanoparticles (*n* = 3).

The synthesis pathway for LPC-TCZ NPs is depicted in Figure 2A. Firstly, the LPC core was synthesized using the inverse microemulsion method followed by the thin-film dispersion method to obtain LPCs. Finally, TCZ was immobilized onto the surface of the nanoparticle using the EDC/NHS method. The TEM images of the LPC core and LPC-TCZ NPs show a uniform spherical shape with an evident phospholipid layer on the outer surface (Figure 2B–E). The successful encapsulation of cisplatin in the NPs was confirmed by the uniform distribution of platinum and chlorine elements inside the LPC core, as revealed by elemental mapping (Figure 2F). The LPCs and LPC-TCZ NPs had an average particle diameter of 317.77 ± 18.09 nm and 318.63 ± 12.85 nm, respectively, determined using dynamic light scattering. The zeta potential and polydispersity index of the LPCs were −8.58 ± 0.28 mV and 0.219 ± 0.036, while those of LPC-TCZ NPs were −6.36 ± 0.88 mV and 0.221 ± 0.019, respectively, indicating a good dispersion of the nanoparticles (Table 1). The successful coupling of TCZ with the surface of lipid-coated cisplatin nanoparticles was verified using SDS-PAGE gel electrophoresis and Coomassie blue staining. LPCs and LPC-TCZ NPs showed white lipids between 10 and 15 kDa (Figure 2G), and both free TCZ and LPC-TCZ had two protein bands around 25 and 55 kDa, indicating the successful connection of TCZ to the surface of LPC-TCZ NPs. The encapsulation rate of CDDP and the coupling efficiency of TCZ were 31.13% and 66.17%, respectively (Table 2). The release behavior of CDDP from LPC-TCZ NPs was investigated using the dynamic dialysis method in PBS at 37 °C, and the release rate was slow, with less than 50% of CDDP being released after 48 h (Figure 2H), indicating the long-term cycling stability of LPC-TCZ NPs.

### 3.3. Cellular Uptake and Cytotoxicity of LPC-TCZ NPs

IL-6, a protein consisting of 184 amino acids with a molecular weight of 26 kDa, is often overexpressed and secreted by different tumor cells and tumor-related cells into the tumor microenvironment [35]. Upon binding to its receptor (IL-6R), IL-6 induces gp130 dimerization and activates downstream signaling pathways, including the JAK/STAT, MAPK, and PI3K/AKT pathways [36,37], thereby promoting tumor resistance. Specifically, studies have shown that IL-6 activates the transcription factors C/EBP-β and C/EBP-δ via the gp130/MAPK/STAT3 signaling pathway, leading to the upregulation of the multidrug-resistant protein P-glycoprotein and promoting drug resistance in breast cancer cells [38]. In addition, IL-6 has been linked to drug resistance in cisplatin and paclitaxel treatments. For instance, IL-6 can upregulate drug-resistant proteins, such as MDR1 and glutathione S transferase pi, downregulate caspase 3, upregulate survival proteins XIAP, Bcl-2, and Bcl-xL, and reduce the toxicity of paclitaxel and cisplatin, thereby protecting tumor cells [39,40]. Studies have also demonstrated that camptothecin can induce the secretion of IL-6 in lung cancer cells by activating the ataxic telangiectasia/p38/NF-κB signaling pathway, which subsequently upregulates the multidrug-resistant protein ABCG2 and anti-apoptotic proteins Bcl-2 and Bcl-xL, leading to drug resistance in tumor cells [41,42]. In addition, IL-6 has been found to promote tumor cell metastasis by promoting the epithelial-to-mesenchymal transition (EMT) process. This process is characterized by decreased expression of E-cadherin and an increased expression of N-cadherin, vimentin, Snail, and Twist, resulting in the loss of epithelial cell characteristics and the transition to mesenchymal cell characteristics [19,43]. In small-cell lung cancer, IL-6 activates downstream STAT3 by activating the CCL2/CCR2 signaling pathway, which reduces the expression of E-cadherin and increases the expression of N-cadherin, thereby promoting the EMT process [19,44,45]. Notably, treatment with recombinant IL-6 has been observed to increase the intracellular expression of Vimentin and Snail, as well as decreasing the expression of E-cadherin in A549 and HCC827 cells. However, this process can be reversed by metformin via the activation of STAT3 [46]. Moreover, IL-6 can enhance the stem cell properties of tumor cells, which can lead to drug resistance and tumor recurrence [47]. IL-6 promotes DNA hypermethylation and inhibits p53 and p21 by activating DNA methyltransferase 1, thus increasing the number of lung cancer stem cells [48,49]. In addition, myofibroblasts of bone marrow origin can promote the growth of colon cancer stem cells by activating the IL-6/JAK2/STAT3 signaling pathway. Furthermore, damage to p53 in basal-like breast cancer can lead to overexpression of IL-6 and inhibition of the methylation of CD44 and CD133 proximal promoters, thereby promoting the expression of stem cell markers CD44 and CD133 [50,51].

**Figure 3:**
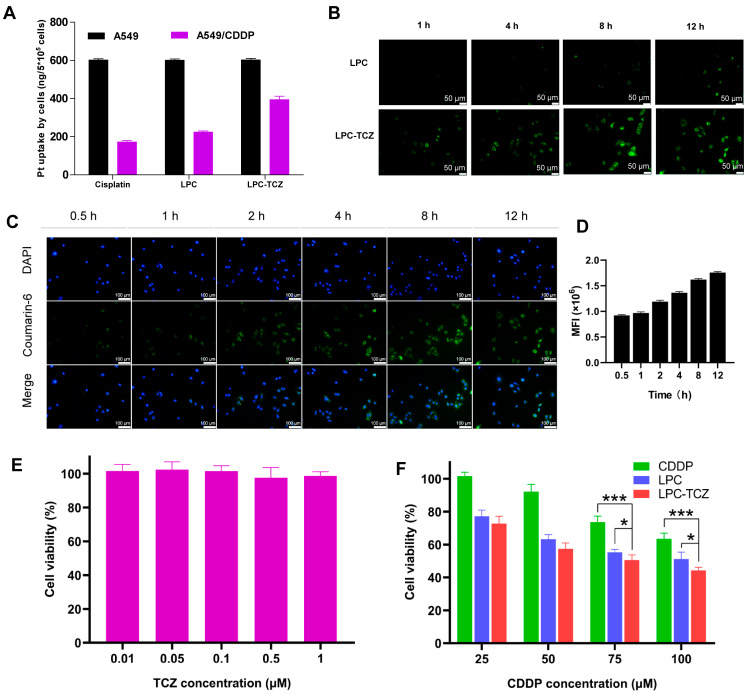
In vitro cellular uptake and anti-tumor effects of particles. (**A**) Uptake of free cisplatin, LPC, and LPC-TCZ by A549 and A549/CDDP cells at 48 h. (**B**) Images from fluorescence microscopy display the absorption of LPC and LPC-TCZ at various time intervals (1 h, 4 h, 8 h, and 12 h). (**C**,**D**) Cellular uptake of LPC-TCZ/coumarin 6 by A549/CDDP cells. (**E**) Effects of TCZ on the proliferation of A549/CDDP cells. (**F**) Effects of CDDP, LPC, and LPC-TCZ on the proliferation of A549/CDDP cells (*n* = 5; * *p* < 0.05; *** *p* < 0.001).

In NSCLC, chemotherapy resistance is a leading reason for treatment failure after several treatment cycles. A key factor in chemotherapy resistance in NSCLC cells is the decreased absorption and concentration of chemotherapeutic drugs in tumor cells that are resistant to treatment [7,52]. In order to examine the uptake of nanoparticles in drug-resistant NSCLC cells, we administered free cisplatin, lipid–polymer hybrid nanoparticles, and LPC-TCZ to both standard A549 cells and cisplatin-resistant A549 cells (A549/CDDP). The concentration of platinum (Pt) was subsequently quantified utilizing inductively coupled plasma mass spectrometry (ICP-MS). Our findings showed that the Pt concentration in A549 cells treated with free cisplatin, LPC, and LPC-TCZ was comparable, measuring approximately 600 ng per 5 × 10^5^ cells. Conversely, in A549/CDDP cells, the Pt concentration was observed to be 175, 213, and 395 ng per 5 × 10^5^ cells, respectively (Figure 3A). The findings indicate that using nanoparticles for drug delivery could potentially overcome chemotherapy resistance in NSCLC cells. Our study further demonstrated that A549/CDDP cells exhibited a reduced uptake of cisplatin and LPC; however, this effect was undone when treated with LPC-TCZ. We performed an experiment with coumarin 6-labeled LPC and LPC-TCZ to evaluate uptake efficiency. Fluorescence microscopy showed that LPC-TCZ had much higher fluorescence intensity than LPC, indicating LPC-TCZ’s good active targeting ability (Figure 3B). Green fluorescence appeared in almost all cells at 0.5 h, suggesting that LPC-TCZ could be rapidly taken up by tumor cells. Subsequently, the fluorescence intensity in the tumor cells gradually increased, reaching its maximum at 12 h (Figure 3C,D). In order to verify that TCZ can reverse CDDP resistance, we performed an MTT assay on A549/CDDP cells. Within the concentration range of 0.01–1 μM, TCZ had almost no killing effect on tumor cells (Figure 3E). As illustrated in Figure 3F, the cytotoxic efficacy of LPCs on A549/CDDP cells surpasses that of free cisplatin when administered at equivalent concentrations. When the CDDP concentration was 75 μM, the cell viability of LPC-TCZ nanoparticles was 4.67% lower than that of LPCs, and when the CDDP concentration was 100 μM, the cell viability of LPC-TCZ nanoparticles was 6.86% lower than that of LPCs. These findings suggest that TCZ may enhance the responsiveness of tumor cells to CDDP.

### 3.4. Synergistic Anti-Tumor Mechanism of LPC-TCZ NPs in A549/CDDP

**Figure 4:**
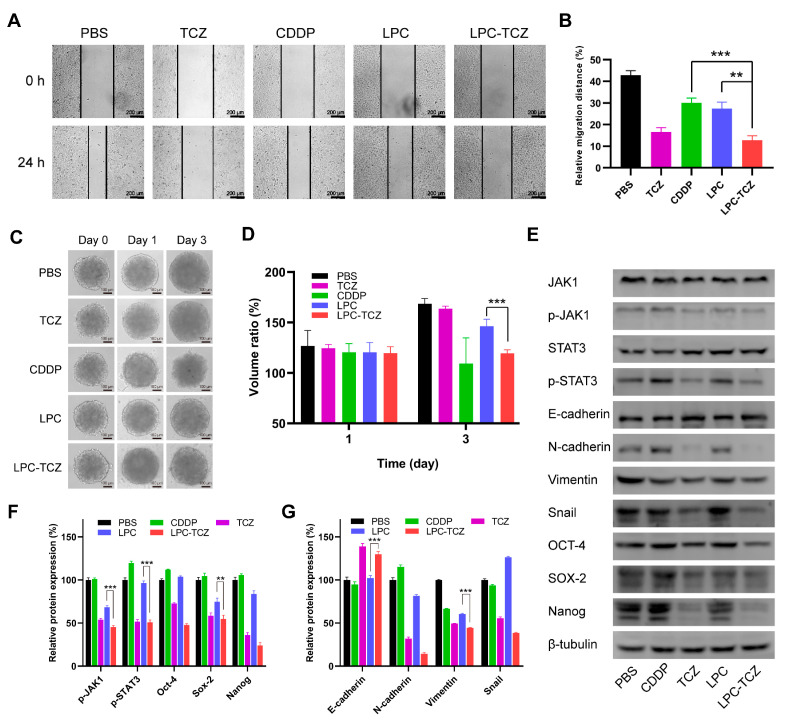
Investigation into the mechanisms by which LPC-TCZ reverses cisplatin resistance through IL-6 regulation in A549/CDDP cells. (**A**) Representative images of the wound healing assay at 0 h and 24 h. (**B**) Measurement of the cell migration distance. (**C**) Representative images of MCTSs treated with different drugs. (**D**) Quantification of the growth of MCTSs treated with different drugs. (**E**) Western blot analysis of the IL-6 signaling pathway and EMT-related proteins. (**F**) Western blot analysis of cancer stem cell markers. (**G**) Quantification of the expression of cancer stem cell markers by the signal intensity of protein bands (*n* = 3; ** *p* < 0.01; *** *p* < 0.001).

A wound healing assay was conducted to investigate the anti-metastatic effects of LPC-TCZ NPs. As depicted in Figure 4A, both CDDP and TCZ demonstrated the ability to impede cell motility. However, a more potent inhibition of wound healing was observed in groups that contained TCZ compared to CDDP. The cell migration rates were 27.36% and 12.71% for the LPC group and LPC-TCZ group, respectively (Figure 4B), indicating significantly enhanced inhibition of cell migration by nanoparticles coupled with TCZ. Furthermore, the inhibitory effect of LPC-TCZ NPs on MCTS growth was evaluated. As illustrated in Figure 4C,D, LPCs exhibited minimal effects on MCTS growth, likely due to inadequate drug release and penetration. On day 3, the volume of MCTSs in the LPCs group had increased to 146%, while the volume of MCTSs in the LPC-TCZ group had only increased to 119%, indicating that TCZ can effectively enhance the inhibitory effect of CDDP on the growth of MCTSs.

As previously mentioned, the gp130/JAK/STAT3 pathway is the primary signaling pathway of IL-6, which can regulate the EMT process and stem cell properties of tumors. During the EMT process, E-cadherin is downregulated, while N-cadherin, vimentin, and Snail are upregulated, leading to reduced intercellular adhesion and increased cell movement and migration. In addition, tumor-resistant cells often overexpress Oct-4, Sox-2, and Nanog [53]. To measure the changes in EMT-related and CSC-related proteins in A549/CDDP cells treated with different drugs, Western blot analysis was conducted. β-tubulin was used as a loading control. As illustrated in Figure 3E,F, the levels of JAK1 and STAT3 did not exhibit significant changes after the A549/CDDP cells were incubated with individual components. However, the expressions of p-JAK1 and p-STAT3 decreased in both the free TCZ and LPC-TCZ NP groups, indicating that TCZ can inhibit the phosphorylation of JAK1 and STAT3. The expression of EMT-related proteins was also measured. Free CDDP and LPCs had minimal regulatory effects on EMT-related proteins, while free TCZ and LPC-TCZ NPs upregulated E-cadherin levels and downregulated N-cadherin, vimentin, and Snail expression (Figure 4E,G), illustrating that TCZ can reverse the EMT process. Furthermore, Figure 4E,F indicate that both TCZ and LPC-TCZ could downregulate the expression of Oct-4, Sox-2, and Nanog, suggesting that TCZ can reduce the expression of cancer stem cell markers. In summary, TCZ can reverse the EMT process and inhibit CSC properties by inhibiting the phosphorylation of JAK1 and STAT3.

The inhibition of tumor stem cell marker expression can decrease the number of stem cell populations within tumors and enhance tumor sensitivity to CDDP, thereby reversing drug resistance. Western blot assays demonstrated that TCZ inhibited the expression of tumor stem cell markers Oct-4, Sox-2, and Nanog, thus elucidating the mechanism behind TCZ’s ability to augment the efficacy of CDDP in the MTT assay and the tumor stem cell ball inhibition assay. However, the effectiveness of TCZ in reversing cisplatin resistance in vitro may be limited because the in vitro action evaluation experiment solely utilized A549/CDDP cells. No other cells within the tumor microenvironment secreted IL-6, while the tumor cells themselves produced limited amounts of IL-6, resulting in an inactive pathway [31]. Moreover, the Western blot results demonstrated that TCZ could reverse the EMT process by upregulating E-cadherin expression and downregulating the expression of N-cadherin, vimentin, and Snail through the inhibition of JAK1 and STAT3 phosphorylation, which explains the mechanism underlying TCZ’s inhibition of cell migration in the scratch experiment.

### 3.5. In Vivo Biodistribution Profiles of LPC-TCZ NPs

**Figure 5:**
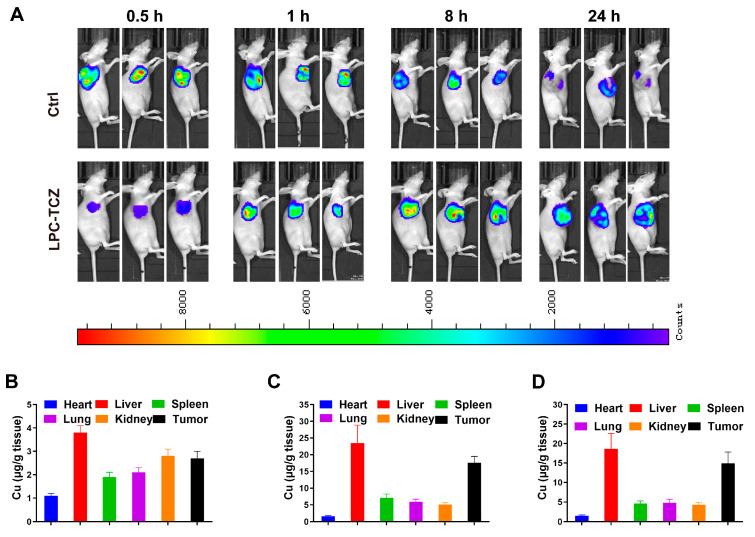
Distributions of LPC-TCZ NPs in an A549/CDDP nude mouse tumor-transplanted model. (**A**) Distribution of LPC-TCZ in tumor tissue detected using a small-animal imaging system. (**B**–**D**) Pt levels in mouse tissues at 0.5, 8, and 24 h following intravenous administration of cisplatin or LPC-TCZ. Data are presented as means ± SEM, with a sample size of *n* = 5.

Animal transplantation tumor assays currently represent the predominant model for screening anti-tumor drugs and conducting oncological research. Notably, the majority of chemotherapeutic agents have been identified through this approach. This model offers several advantages, such as the capacity to inoculate a cohort of animals with a uniform quantity of tumor cells or cell-free filtrate (viral tumor), thereby ensuring a more consistent growth rate, reduced inter-individual variability, and an almost 100% inoculation survival rate. In this research, we developed a nude mouse tumor-transplanted model through in vitro transplantation of A549/CDDP, examining the drug delivery and pharmacodynamics of LPC-TCZ. We investigated drug delivery in tumor-implanted nude mice by utilizing biotin-labeled LPC-TCZ and monitored its distribution within tumor tissues over various time intervals. The findings indicated that following the administration of LPC-TCZ via the caudal vein, the fluorescence intensity reached its maximum, suggesting that LPC-TCZ circulated within the organism for 8 h before beginning to accumulate at the tumor site (Figure 5A).

Subsequently, we examined how cisplatin was distributed after LPC-TCZ was injected. The experimental results demonstrated that Pt exhibited significant accumulation in tumor tissues within 8 h post-administration, with sustained high Pt concentrations persisting at the tumor sites even after 24 h (Figure 5B–D). These findings collectively indicate that the LPC-TCZ delivery system enables targeted drug transport to neoplastic regions while maintaining prolonged tumoral retention, thereby demonstrating both tumor-targeting specificity and enhanced pharmacokinetic properties through long-circulation effects.

### 3.6. In Vivo Anti-Tumor Activity

**Figure 6:**
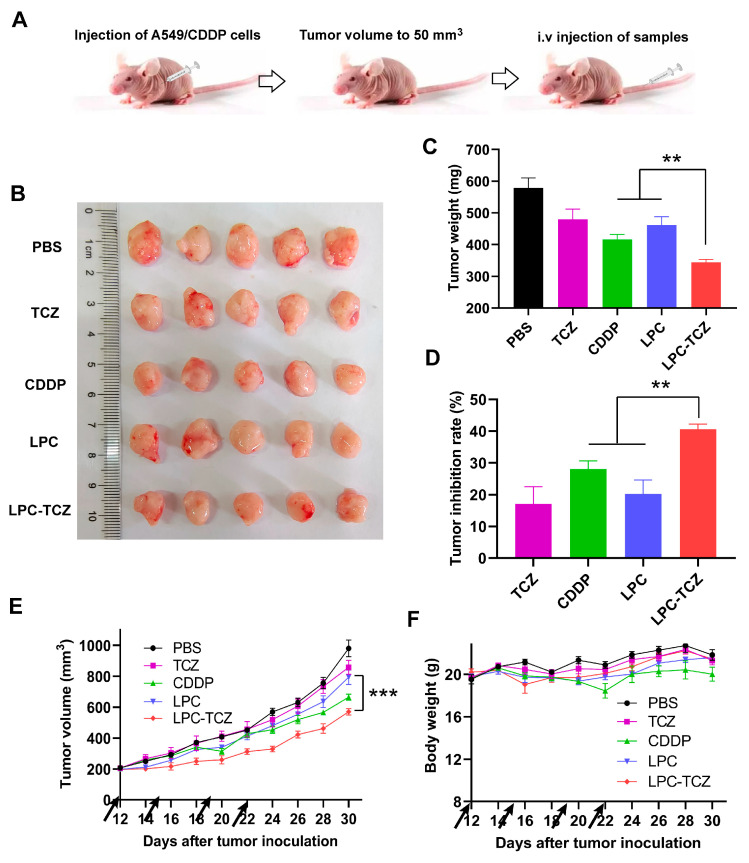
Anti-tumor effects of LPC-TCZ in a xenograft mouse model (equivalent to 1 mg/kg cisplatin). (**A**) Schematic diagram of the experimental procedure. (**B**) Image of excised tumors at the end of the experiment. (**C**) Tumor weight of each experimental group at the end of the experiment. (**D**) Tumor inhibition rate of each experimental group at the end of the experiment. (**E**) Changes in tumor volume during treatment. (**F**) Changes in body weight during treatment. The arrow indicates the time of drug administration. The data presented are the means ± standard deviation, with a sample size of *n* = 5. ** *p* < 0.01; *** *p* < 0.001.

**Figure 7:**
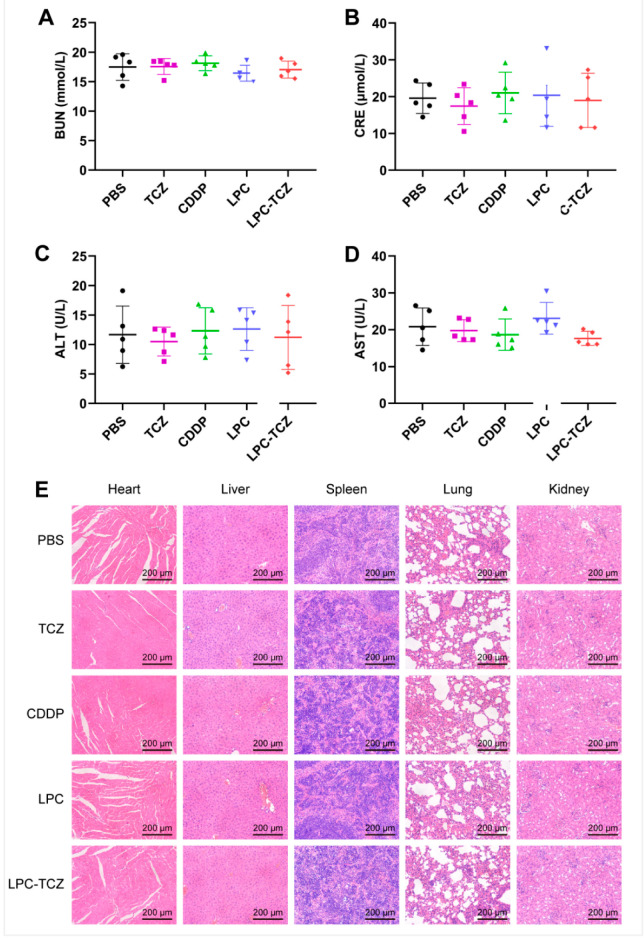
Levels of liver and kidney indexes—BUN (**A**), CRE (**B**), ALT (**C**), and AST (**D**)—in serum at the end of the experiment (*n* = 5). (**E**) H&E-stained images of heart, liver, spleen, lung, and kidney at the end of the experiment. The data presented are the means ± standard deviation.

The in vivo anti-tumor effect of LPC-TCZ nanoparticles (NPs) was evaluated using the A549/CDDP xenograft model. Saline, TCZ, free cisplatin, LPC, and LPC-TCZ were further assessed in a nude mice model bearing A549/CDDP xenografts. Pharmacological intervention commenced with four doses once the tumor volume had attained a size of 50–100 mm^3^. On the final day of the study, the mice were euthanized, and tumor specimens were collected for analysis (Figure 6B). As depicted in Figure 4C, the tumor volume had reached approximately 200 mm^3^ in BALB/c nude mice 12 days post-tumor inoculation. Different drugs were administered via the tail vein on days 12, 15, 19, and 22. BALB/c nude mice were euthanized on day 30, and blood, tumors, and major organs were collected. As illustrated in Figure 6C,E, the tumors in the PBS group were the largest on day 30, with an average weight of 579.16 mg. TCZ, cisplatin, and LPC NPs inhibited tumor growth. The average tumor weight of the LPC-TCZ NP group was the lowest, at 344.00 mg. Correspondingly, LPC-TCZ NPs had the best tumor-inhibitory effect, with a tumor-inhibition rate of 40.60% (Figure 6D), suggesting that TCZ could enhance the toxicity of cisplatin to tumors. As depicted in Figure 6F, the body weight of all BALB/c nude mice fluctuated around 20 g after treatment with different drugs. On day 22, the body weight of BALB/c nude mice in the cisplatin group had decreased significantly, and the body weight gradually decreased after stopping tail vein injection, indicating that free cisplatin had a certain toxicity. The body weight of BALB/c nude mice in the LPC and LPC-TCZ NP groups did not significantly decrease, indicating that nanoparticles could reduce the toxicity of cisplatin to normal tissues. The results of serum liver and kidney indexes of BALB/c nude mice and H&E-stained sections of major organs demonstrated that LPCs and LPC-TCZ NPs had no significant liver or kidney toxicity (Figure 7), indicating that the nanoparticles had good biocompatibility, which was consistent with the weight changes.

The results of the transplanted tumor experiment showed that TCZ alone exhibited certain anti-tumor effects, suggesting that TCZ can inhibit tumor growth by suppressing the IL-6 signaling pathway, which is consistent with previous reports in the literature. However, the previous MTT experiment demonstrated that TCZ could not effectively inhibit cell proliferation in vitro within the concentration range of 0.01–1 μM. This may be due to the fact that in vitro experiments only involved tumor cells that secreted a small amount of IL-6; however, in in vivo experiments, a tumor model was established and mesenchymal stem cells, tumor-related fibroblasts, and macrophages in the tumor microenvironment secreted IL-6 to act on tumor cells and promote their proliferation. Moreover, the anti-tumor effect of LPC-TCZ was stronger than that of LPC and CDDP, indicating that TCZ can also reverse cisplatin resistance in vivo, which is consistent with the results of the MTT experiment and the tumor stem cell ball growth inhibition experiment described above.

### 3.7. Synergistic Anti-Tumor Mechanism of LPC-TCZ NPs in Tumor Tissue

**Figure 8:**
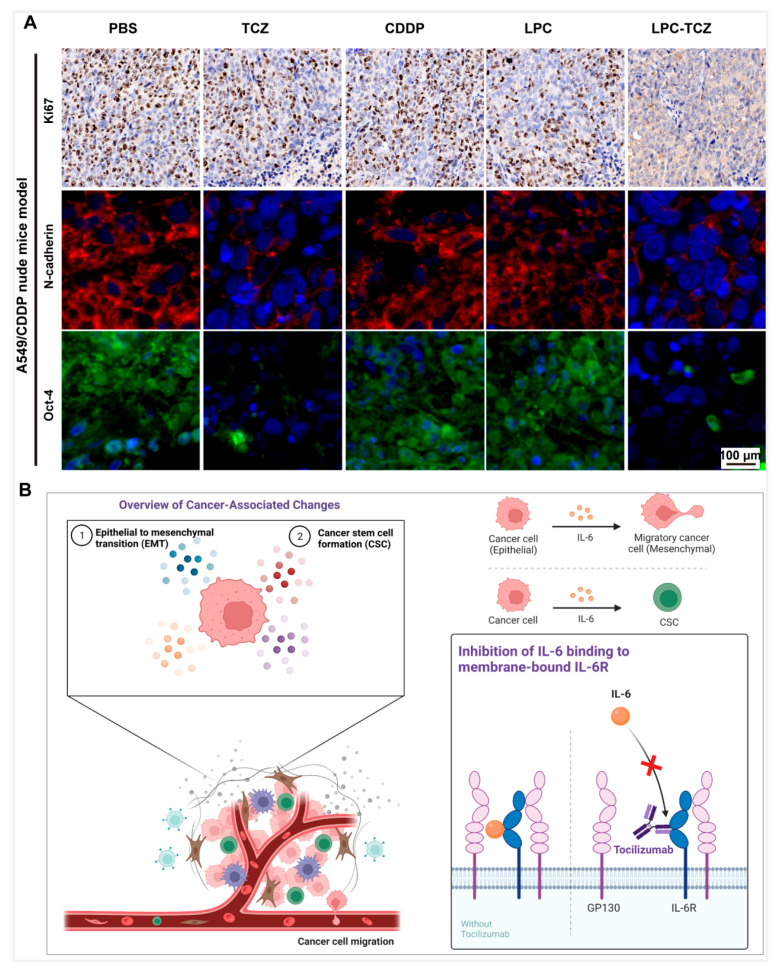
Mechanisms underlying the reversal of cisplatin resistance via LPC-TCZ through the regulation of IL-6 in tumor tissues of xenograft mouse models. (**A**) Fluorescent imaging of immunohistochemical markers Ki67, N-cadherin, and Oct-4 in tumor tissues. The Ki67 maker is shown in brown, N-cadherin in green, and Oct-4 in red, while the nucleus is shown in blue. (**B**) Schematic illustrating the anti-tumor mechanisms of LPC-TCZ NPs in NSCLC. IL-6 regulates tumor EMT processes and stem cell properties by inducing gp130 dimerization and promoting JAK1 and STAT3 phosphorylation. By binding with IL-6R, TCZ enhances the expression of E-cadherin while simultaneously reducing the expression of N-cadherin, vimentin, and Snail. This modulation effectively reverses the EMT process and inhibits cellular migration. Additionally, TCZ reduces the number of stem cell populations in tumor cells by inhibiting the expression of tumor stem cell markers Oct-4, Sox-2, and Nanog, as well as enhancing the inhibitory effect of CDDP on tumor stem cells, thus improving the efficacy of CDDP.

The studies mentioned above demonstrated that LPC-TCZ can hinder the proliferation of drug-resistant NSCLC and prevent the CSC and EMT of NSCLC. To elucidate the effects of LPC-TCZ on CSC and EMT signaling, tumor tissues were observed. The main indicators of changes in NSCLC tissues were Ki67, a marker of tumor cell proliferation, N-cadherin, an EMT target, and oct-4, a recognized marker of NSCLC CSCs in recent years. Immunofluorescence staining analysis indicated a significant reduction in EMT (N-cadherin) and NSCLC CSC (oct-4) signaling in the A549/CDDP nude mouse model following treatment with TCZ and LPC-TCZ. However, the effect of TCZ on proliferation (Ki67) was not prominent, indicating that TCZ played a role in enhancing cisplatin and had a multiplier effect (Figure 8A). These results indicate that LPC can enhance the inhibition of CSC formation and the AMT process of NSCLC through the combination of TCZ in a nude mouse tumor transplanted model.

In conclusion, we successfully prepared lipid-coated cisplatin nanoparticles coupled with TCZ, which reversed CDDP resistance and inhibited tumor cell metastasis by inhibiting the IL-6 signaling pathway, thus enhancing the therapeutic effect of CDDP (Figure 8B). However, there are still some limitations in the method and content of this study. Firstly, this study only discussed the role of TCZ from the perspective of EMT and tumor stem cell characteristics. However, the IL-6 signaling pathway is complex, and its downstream signaling pathways also include PI3K/AKT and MAPK, which are related to tumor resistance. Additionally, IL-6 is involved in multiple processes of tumor development, such as activating other tumorigenic factors and promoting angiogenesis. Whether TCZ can inhibit these signaling pathways or tumor progression requires further investigation. Secondly, the encapsulation rate of CDDP needs improvement, possibly because of the fact that during the preparation of DOPA-coated CDDP nanoparticles using the reverse-phase microemulsion method, some cisplatin prodrugs react with KCl to form CDDP precipitation, which is not coated by DOPA. Consequently, phospholipids cannot be coated in the membrane hydration process. The next step involves optimizing the preparation process of nanoparticles to improve the encapsulation rate of CDDP, such as increasing the DOPA dosage or changing the oil-phase ratio.

## 4. Conclusions

In this study, our objective was to develop a targeted nanoplatform to address cisplatin resistance in NSCLC by modulating the IL-6 signaling pathway. To this end, we successfully synthesized LPC-TCZ. Through extensive in vitro and in vivo investigations, we demonstrated that LPC-TCZ effectively inhibited tumor growth, diminished CSC markers, suppressed EMT, and enhanced cisplatin sensitivity in resistant NSCLC models. Mechanistically, LPC-TCZ exerts its therapeutic effects by obstructing the IL-6/gp130/JAK/STAT3 signaling cascade, thereby reversing EMT and CSC phenotypes. We further validated the altered expression of EMT- and CSC-associated proteins, including E-cadherin, N-cadherin, Snail, vimentin, Oct-4, and Sox-2, in cisplatin-resistant NSCLC cells. Collectively, our findings suggest that LPC-TCZ possesses significant potential as a dual-function therapeutic strategy targeting both chemoresistance and metastasis in NSCLC.

The LPC-TCZ nanoplatform developed in this study shows the potential to overcome cisplatin resistance and inhibit metastasis in NSCLC. However, its long-term safety, pharmacokinetics, and in vivo stability need further investigation. In addition, tumor heterogeneity may affect its effectiveness in different patients. Future studies will aim to improve the formulation, test it in patient-derived models, and explore combinations with immunotherapy to support clinical translation.

## Figures and Tables

**Table 1:** Particle size, zeta potential, and PDI of nanoparticles.

	Particle Size (nm)	Zeta Potential (mV)	PDI
**LPC**	317.77 ± 18.09	−8.58 ± 0.28	0.219 ± 0.036
**LPC-TCZ**	318.63 ± 12.85	−6.36 ± 0.88	0.221 ± 0.019

**Table 2:** CDDP encapsulation rate and TCZ coupling efficiency.

	Encapsulation Efficiency of CDDP (%)	Coupling Efficiency of TCZ (%)
**1**	32.13	65.92
**2**	33.08	62.86
**3**	28.18	69.72
**Mean ± SD**	31.13 ± 2.60	66.17 ± 3.44

## Data Availability

The data are contained within the article.
